# Effect of Tauroursodeoxycholic Acid on Inflammation after Ocular Alkali Burn

**DOI:** 10.3390/ijms231911717

**Published:** 2022-10-03

**Authors:** Yanqiao Huang, Lixia Lin, Yao Yang, Fang Duan, Miner Yuan, Bingsheng Lou, Xiaofeng Lin

**Affiliations:** State Key Laboratory of Ophthalmology, Guangdong Provincial Key Laboratory of Ophthalmology and Visual Science, Zhongshan Ophthalmic Center, Sun Yat-sen University, Guangzhou 510060, China

**Keywords:** ocular alkali burns, inflammation, tauroursodeoxycholic acid, endoplasmic reticulum stress

## Abstract

Inflammation is the main cause of corneal and retinal damage in an ocular alkali burn (OAB). The aim of this study was to investigate the effect of tauroursodeoxycholic acid (TUDCA) on ocular inflammation in a mouse model of an OAB. An OAB was induced in C57BL/6j mouse corneas by using 1 M NaOH. TUDCA (400 mg/kg) or PBS was injected intraperitoneally (IP) once a day for 3 days prior to establishing the OAB model. A single injection of Infliximab (6.25 mg/kg) was administered IP immediately after the OAB. The TUDCA suppressed the infiltration of the CD45-positive cells and decreased the mRNA and protein levels of the upregulated TNF-α and IL-1β in the cornea and retina of the OAB. Furthermore, the TUDCA treatment inhibited the retinal glial activation after an OAB. The TUDCA treatment not only ameliorated CNV and promoted corneal re-epithelization but also attenuated the RGC apoptosis and preserved the retinal structure after the OAB. Finally, the TUDCA reduced the expression of the endoplasmic reticulum (ER) stress molecules, IRE1, GRP78 and CHOP, in the retinal tissues of the OAB mice. The present study demonstrated that the TUDCA inhibits ocular inflammation and protects the cornea and retina from injury in an OAB mouse model. These results provide a potential therapeutic intervention for the treatment of an OAB.

## 1. Introduction

An ocular alkali burn (OAB) is one of the most severe ophthalmic emergencies and is responsible for 6.9% to 13.2% of ocular injuries [1,2]. The majority of affected individuals are young males of working age in developing countries [3]. Nearly 20% of patients are considered legally blind, even after appropriate treatment [4,5]. The main reason for a poor visual prognosis is the severe and persistent inflammatory response after an OAB, which can promote corneal neovascularization (CNV), impair corneal re-epithelization, and even damage the retinal ganglion cells (RGCs) [2,6]. It is well known that conventional OAB treatments usually focus on ocular surface reconstruction [7]. However, treatments that can attenuate both anterior and posterior segment injuries following an OAB are limited.

The corneal damage in an OAB is characterized by an aggressive inflammatory response, including massive leukocyte infiltration and excessive inflammatory cytokines release. Multiple studies have reported that inflammation is an important mechanism of CNV [8]. In the worst case, corneal perforation occurs due to excessive inflammation and impaired re-epithelialization [9]. Moreover, several recent studies demonstrated that an OAB can affect the retina and optic nerve. The mechanism of retinal damage after an OAB is also closely related to inflammation, manifested by the increased expression of destructive inflammatory cytokines and the activation of immune cells in the retina [6,10,11,12,13]. Zhou et al. found that an anti-TNF-α (infliximab) treatment could alleviate the extensive damage to the cornea, retina, and optic nerve in OAB eyes [13]. However, the systemic administration of infliximab could have some serious side effects [14]. Therefore, there is an urgent need for an effective and safe therapeutic approach to reduce OAB-induced ocular inflammation.

In our previous study, we found that endoplasmic reticulum (ER) stress participates in the retinal damage after an OAB. The inhibition of ER stress suppresses the retinal inflammation and reduces the RGC apoptosis in the OAB model [15]. Tauroursodeoxycholic acid (TUDCA), a chemical chaperone, has been demonstrated to be a common ER stress inhibitor by improving the ER-folding capacity and found to have anti-apoptosis and anti-inflammation effects [16,17,18]. Importantly, TUDCA has been evaluated in clinical trials of some neurodegenerative diseases and has not shown any adverse events during treatment [19,20]. However, it is unclear whether TUDCA inhibits ocular inflammation and has a protective effect on OAB eyes.

The purpose of this study was to examine the anti-inflammatory effect of TUDCA and investigate whether TUDCA exerts a therapeutic effect in a mouse model of an OAB.

## 2. Results

### 2.1. TUDCA Ameliorated Corneal Structural Damage after OAB

**Figure 1:**
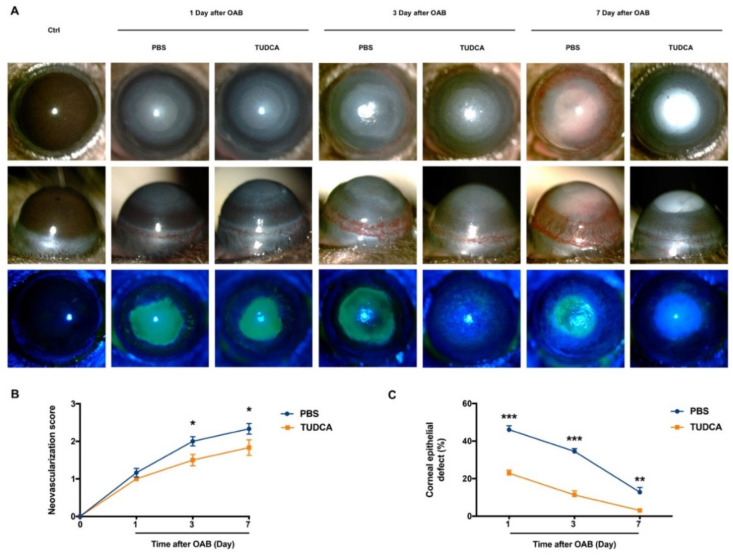
TUDCA ameliorates CNV and promotes corneal re-epithelization after OAB. (**A**) Macroscopic and fluorescein sodium-stained images of control and alkali-burned eyes were taken 1, 3, and 7 days post-injury. (**B**) The scores of CNV in alkali injury eyes treated with PBS or TUDCA at different times. (**C**) Quantitative analysis of corneal epithelial defect area in the PBS-treated group and TUDCA-treated group at different times after OAB. The percentage of epithelial defect area is the area of sodium fluorescein staining in each eye compared to the area of the entire cornea. Data are expressed as mean ± SEM (*n* = 12 mice/group, * *p* < 0.05, ** *p* < 0.01, *** *p* < 0.001). Ctrl, control; OAB, ocular alkali burn; TUDCA, tauroursodeoxycholic acid; CNV, corneal neovascularization.

The OAB model was confirmed in photographs of the anterior segment (Figure 1A). The score of the CNV gradually increased over time after the OAB. The TUDCA treatment significantly reduced the mean scores of the vascularization on day 3 and day 7 after the OAB (*p* *<* 0.05, Figure 1B). A corneal epithelial defect was visualized by fluorescein staining. The results showed that the percentage of the corneal epithelial defect area in the TUDCA-treated group was lower than that in the PBS-treated group at each time point after the OAB (22.85% ± 1.66% versus 46.08% ± 2.07% on day 1, *p* *<* 0.001; 11.32% ± 2.14% versus 34.50% ± 1.48% on day 3, *p* *<* 0.001; and 3.06% ± 0.65% versus 12.78% ± 2.55% on day 7, *p* *=* 0.001; Figure 1C). These findings suggested that TUDCA could ameliorate CNV and promote epithelial repair in alkali-induced corneas.

### 2.2. TUDCA Suppressed Corneal Inflammation after OAB

**Figure 2:**
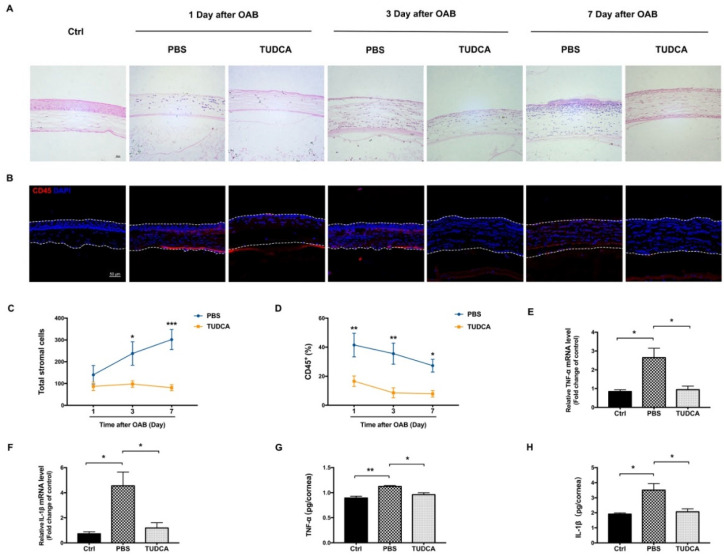
TUDCA suppresses corneal inflammation after OAB. (**A**) H&E staining of corneas in the control group, PBS-treated group, and TUDCA-treated group at 1, 3, and 7 days after OAB. Scale bar: 20 μm. (**B**) Immunofluorescence staining with the leukocyte marker CD45 in the different groups. Scale bar: 50 μm. (**C**) Quantitative analysis of corneal stromal cells in alkali injury eyes treated with PBS or TUDCA at different times (*n* = 6 mice/group). (**D**) The quantification of CD45^+^ cells labeling is calculated as the total number of red cells (CD45^+^ cells) divided by the total number of blue cells (DAPI) in the corneal stroma, and then multiplied by 100 (*n* = 6 mice/group). (**E**,**F**) RT-PCR analysis of the inflammatory cytokines TNF-α and IL-1β in corneas collected 7 days after OAB. (**G**,**H**) Protein levels of TNF-α and IL-1β were analyzed by ELISA. Data are expressed as mean ± SEM (*n* = 3, * *p* < 0.05, ** *p* < 0.01, *** *p* < 0.001). Ctrl, control; OAB, ocular alkali burn; TUDCA, tauroursodeoxycholic acid.

A histological analysis of the cornea revealed a progressive increase in the number of corneal stromal cells after the OAB (Figure 2A). The TUDCA treatment significantly reduced the number of corneal stromal cells on day 3 and day 7 after the OAB compared to the PBS treatment (97.66 ± 15.07 cells/field versus 237.83 ± 53.71 cells/field on day 3, *p* = 0.03; and 80.66 ± 13.68 cells/field versus 302.0 ± 46.20 cells/field on day 7, *p* < 0.001, Figure 2B). To analyze the corneal inflammation triggered by the OAB, we evaluated the CD45^+^ leukocytes infiltrating the cornea (Figure 2C). In the control group, there were nearly no CD45^+^ cells in the corneal stroma. Nevertheless, CD45^+^ cells were detected in the corneal stroma after the OAB induction. As expected, the number of CD45^+^ cells was significantly lower in the TUDCA-treated group than in the PBS-treated group at each time point after the OAB (*p* < 0.05; Figure 2D). Furthermore, the mRNA and protein levels of the TNF-α and IL-1β were markedly increased in the OAB corneas 7 days after the OAB. The treatment with TUDCA significantly reduced the corneal expression of TNF-α and IL-1β after the OAB (*p* < 0.05, Figure 2E–H).

### 2.3. TUDCA Protected the Structure of the Retina in OAB Eyes

**Figure 3:**
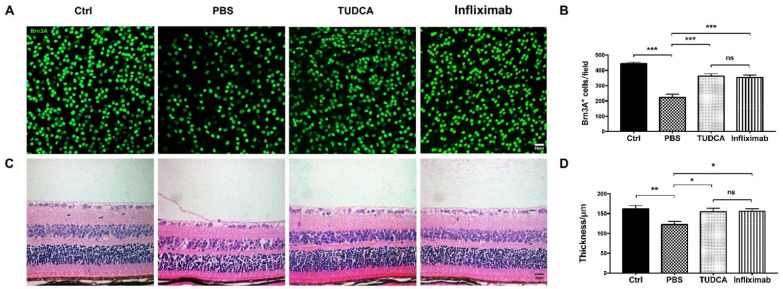
TUDCA preserves the structure of the retina after OAB. (**A**) Representative Brn3A (retinal ganglion cell marker) immunoreactivity in retinal whole mounts from control and alkali burn eyes treated with PBS, TUDCA, or infliximab on day 7 post-injury. (**B**) Quantitative analysis of Brn3A^+^ cells. (**C**) The pictures of the control and injury retinas treated with PBS, TUDCA, or infliximab were taken 7 days after OAB. (**D**) Quantitative analysis of retinal whole thickness. Data are expressed as mean ± SEM (*n* = 8 regions/mouse of six mice, * *p* < 0.05, ** *p* < 0.01, *** *p* < 0.001; ns, no significance). Scale bar: 20 μm. Ctrl, control; OAB, ocular alkali burn; TUDCA, tauroursodeoxycholic acid.

To further confirm whether TUDCA promoted the survival of RGCs in the OAB model, the density of the RGCs in the retinal flat-mounts was compared between the control, PBS-treated, TUDCA-treated, and Infliximab-treated mice on day 7 after the OAB (control: 444.25 ± 9.10 cells/field; OAB with PBS-treated: 222.75 ± 21.31 cells/field; OAB with TUDCA-treated: 361.87 ± 15.43 cells/field; and OAB with Infliximab-treated: 357.37 ± 17.31 cells/field; Figure 3A). The density of the RGCs in the TUDCA-treated and Infliximab-treated groups was significantly higher than in the PBS-treated group (361.87 ± 15.43 cells/field versus 222.75 ± 21.31 cells/field, *p* < 0.001; and 357.37 ± 17.31 cells/field versus 222.75 ± 21.31 cells/field, *p* < 0.001; Figure 3B). There was no significant difference in the RGC density between the TUDCA-treated and Infliximab-treated groups (361.87 ± 15.43 cells/field versus 357.37 ± 17.31 cells/field, *p* > 0.99). These findings show that TUDCA could promote the survival of RGCs in OAB eyes.

The H&E staining was performed to observe the retinal thickness changes in the control, PBS-treated, TUDCA-treated, and Infliximab-treated mice on day 7 after the OAB (control: 161.86 ± 8.52 μm; OAB with PBS-treated: 122.15 ± 8.15 μm; OAB with TUDCA-treated: 154.84 ± 8.63 μm; and OAB with Infliximab-treated: 155.52 ± 6.82 μm; Figure 3C). There was a significant decrease in the overall retinal thickness 7 days after the OAB as compared to the control (122.15 ± 8.15 μm versus 161.86 ± 8.52 μm, *p* = 0.0099, Figure 3D). The treatment with TUDCA or Infliximab significantly increased the retinal thickness following exposure to the alkali injury (154.84 ± 8.63 μm versus 122.15 ± 8.15 μm, *p* = 0.0468; and 155.52 ± 6.82 μm versus 122.15 ± 8.15 μm, *p* = 0.0405; Figure 3D).

### 2.4. TUDCA Attenuated RGC Apoptosis after OAB

**Figure 4:**
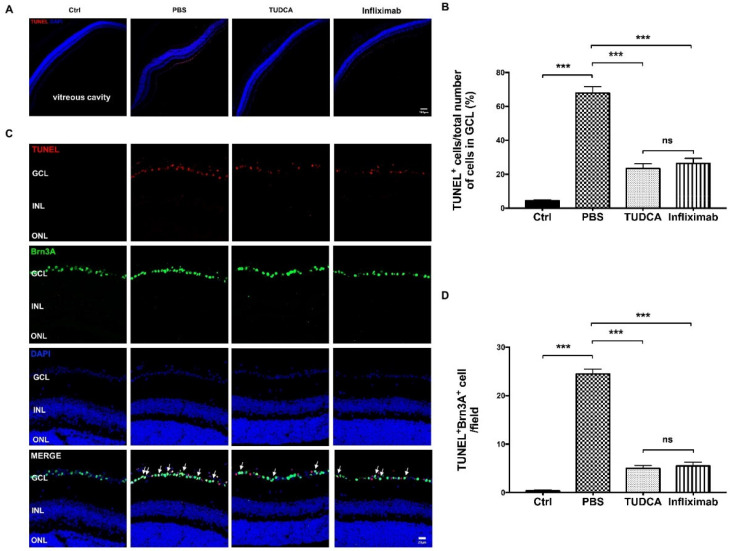
TUDCA attenuates RGC apoptosis after OAB. (**A**) Representative retinal cryosection images stained with TUNEL (red fluorescence) and DAPI (blue) in the control, OAB with PBS-treated, OAB with TUDCA-treated, and OAB with infliximab-treated groups at 1 day after OAB. Scale bar: 100 μm. (**B**) The quantification of TUNEL labeling is calculated as the total number of red cells (TUNEL) divided by the total number of blue cells (DAPI) in the GCL layer, and then multiplied by 100 (*n* = 8 regions/mouse of six mice). (**C**) Double immunofluorescence staining with TUNEL (red fluorescence) and the RGC marker Brn3A (green fluorescence) in different groups. Scale bar: 20 μm. (**D**) Quantitative analysis of TUNEL^+^ Brn3A^+^ cells in each retina area (*n* = 6 mice/group). Data are expressed as mean ± SEM (*** *p* < 0.001; ns, no significance). Ctrl, control; OAB, ocular alkali burn; GCL, ganglion cell layer; TUDCA, tauroursodeoxycholic acid.

We compared the number of apoptotic RGCs between the control, OAB with PBS-treated, OAB with TUDCA-treated, and OAB with Infliximab-treated groups on day 1 after the OAB induction (Figure 4A). In the TUDCA-treated and Infliximab-treated eyes, the number of TUNEL^+^ cells in the ganglion cell layer (GCL) were significantly lower than that in the PBS-treated group (23.46 ± 2.73% versus 67.81 ± 3.84%, *p* < 0.001; and 26.48 ± 2.92% versus 67.81 ± 3.84%, *p* < 0.001) (Figure 4B). There was no significant difference in the number of apoptotic cells in the GCL between the TUDCA-treated and Infliximab-treated groups. Double immunofluorescence labeling was used to confirm the RGC injury after the OAB induction (Figure 4C). The control retina contained only Brn3A^+^ cells; TUNEL^+^ cells were not detected. Nevertheless, there was a significant increase in Brn3A^+^ TUNEL^+^ cells in the OAB retinas compared with the control. Treatment with TUDCA or Infliximab could reduce the number of apoptotic RGCs (*p* *<* 0.001, Figure 4D).

### 2.5. TUDCA Reduced the Retinal Inflammation after OAB

**Figure 5:**
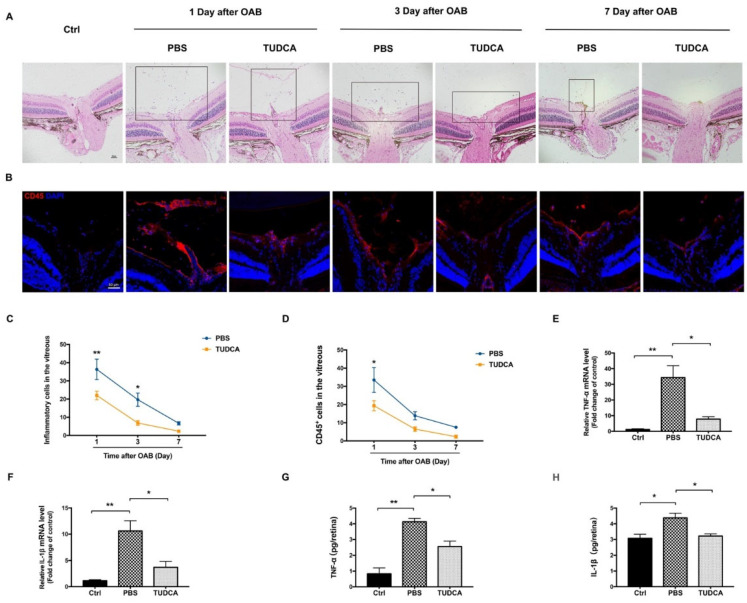
TUDCA reduces the retinal inflammation after OAB. (**A**) H&E staining of posterior segment of eyes in the control group, PBS-treated group, and TUDCA-treated group at 1, 3, and 7 days after OAB. (**B**) CD45 immunolocalization in different groups of retinas. (**C**) Quantitative analysis of inflammatory cells in the vitreous cavity in alkali injury eyes treated with PBS or TUDCA at different times (*n* = 6 mice/group). (**D**) Quantitative analysis of CD45^+^ cells in the vitreous cavity (*n* = 6 mice/group). (**E**,**F**) RT-PCR analysis of the inflammatory cytokines TNF-α and IL-1β in retinas harvested 1 day after OAB. (**G**,**H**) Protein levels of TNF-α and IL-1β were analyzed by ELISA. Data are expressed as mean ± SEM (*n* = 3, * *p* < 0.05, ** *p* < 0.01). Scale bar: 50 μm. Ctrl, control; OAB, ocular alkali burn; TUDCA, tauroursodeoxycholic acid.

To further investigate whether TUDCA reduced the retinal inflammation, H&E staining was performed to study the infiltration of the inflammatory cells in the vitreous cavity after the OAB (Figure 5A). One day after the OAB, a large number of inflammatory cells infiltrated from the optic nerve into the posterior vitreous space, and then the number of inflammatory cells in the vitreous cavity was gradually decreased. The TUDCA inhibited the infiltration of the inflammatory cells in the vitreous cavity caused by the OAB (22.0 ± 2.35 cells/field versus 36.33 ± 5.61 cells/field on day 1, *p* *=* 0.006; 6.83 ± 1.24 cells/field versus 19.66 ± 3.61 cells/field on day 3, *p* *=* 0.01; and 2.33 ± 0.71 cells/field versus 6.66 ± 0.88 cells/field on day 7, *p =* 0.93; Figure 5B). Additionally, the presence of inflammatory cells in the eye sections was assessed by immunofluorescence staining with CD45. As shown in Figure 5C,D, the number of CD45^+^ cells in the posterior vitreous cavity were significantly increased 1 day after the OAB, whereas it was decreased after the TUDCA treatment (33.5 ± 6.81 cells/field versus 19.33 ± 2.74 cells/field, *p =* 0.01). We further investigated the effect of TUDCA on the expression levels of the inflammatory cytokines TNF-α and IL-1β in the retinal tissues on day 1 post-injury. The RT-PCR analysis showed that the TUDCA treatment significantly reduced the mRNA expression of TNF-α and IL-1β after the OAB (Figure 5E,F). The protein levels of TNF-α and IL-1β in the PBS-treated OAB retinal tissues were found to be significantly higher than those in the control retinas and were significantly decreased after treatment with TUDCA (*p* < 0.05; Figure 5G,H).

### 2.6. TUDCA Alleviated Neuroinflammation Induced by OAB

**Figure 6:**
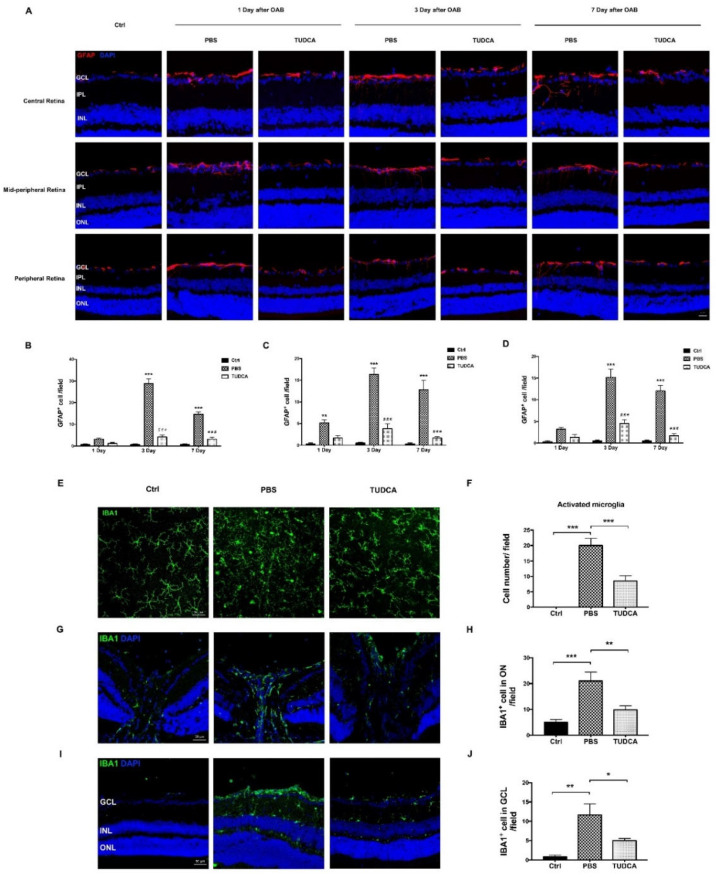
TUDCA alleviates neuroinflammation induced by OAB. (**A**) Representative images of immunostaining, retinal frozen sections from control, and alkali injury eyes treated with PBS or TUDCA after 1, 3, and 7 days were stained with GFAP. Scale bar: 20 μm. (**B**–**D**) Bar chart shows the numbers of GFAP^+^ fibers crossing the IPL and INL at the central retina, mid-peripheral retina, and peripheral retina, respectively. These data are mean ± SEM (*n* = 6 mice/group, ** *p* < 0.01, *** *p* < 0.001 versus the control group; ### *p* < 0.001 versus the PBS-treated group). (**E**) Immunofluorescence staining with IBA1 in retinal whole mounts in the control group, PBS-treated group, and TUDCA-treated group on day 7 post-injury. Scale bar: 50 μm. (**F**) Quantitative analysis of the number of ameboid microglia per field in different groups. (**G**) Immunofluorescence staining with IBA1 in the optic nerve. Scale bar: 50 μm. (**H**) Quantitative analysis of the number of IBA1^+^ cells per section in different groups. (**I**) Microglia activation was assessed in retinal sections by labeling with IBA1 from control eyes and alkali injury eyes treated with PBS or TUDCA. Scale bar: 50 μm. (**J**) Quantitative analysis of the number of IBA1^+^ cells in the GCL per section in different groups. These data are mean ± SEM (*n* = 6 mice/group, * *p* < 0.05, ** *p* < 0.01, *** *p* < 0.001). Ctrl, control; OAB, ocular alkali burn; TUDCA, tauroursodeoxycholic acid; GCL, ganglion cell layer; IPL, inner plexiform layer; INL, inner nuclear layer; ON, optic nerve.

To observe the effect of TUDCA on neuroinflammation, we examined whether the TUDCA inhibited the retinal glial activation in the retina. GFAP (for macroglia) and IBA-1 (for microglia) immunofluorescence were used to determine the glial reactivity in the retina and optic nerve (ON) of the control and OAB eyes. The confocal results showed that the number of GFAP^+^ glial cells in the inner plexiform layer (IPL) and inner nuclear layer (INL) was dramatically increased in the central, mid-peripheral, and peripheral retina after the OAB, peaking on day 3 (*p* < 0.001, Figure 6A–D). The retinal flat mounts showed that the microglia were activated after the OAB, manifested as the morphology of the retinal microglia changing from the normal ramified to amoeboid form (Figure 6E). As depicted in the retinal sections, the number of IBA1^+^ microglia in the ON and GCL was increased on day 7 after the OAB (ON: 21.0 ± 3.44 cells/field versus 5.0 ± 1.06 cells/field, *p =* 0.0005; and GCL: 11.67 ± 2.83 cells/field versus 0.83 ± 0.40 cells/field, *p =* 0.0012; Figure 6G–J). The TUDCA treatment significantly reduced the number of GFAP^+^ glial cells and amoeboid microglia and attenuated the microglia migration to the GCL compared with the PBS-treated group (Figure 6D,F–H).

### 2.7. TUDCA Inhibited ER Stress in OAB Retinas

**Figure 7:**
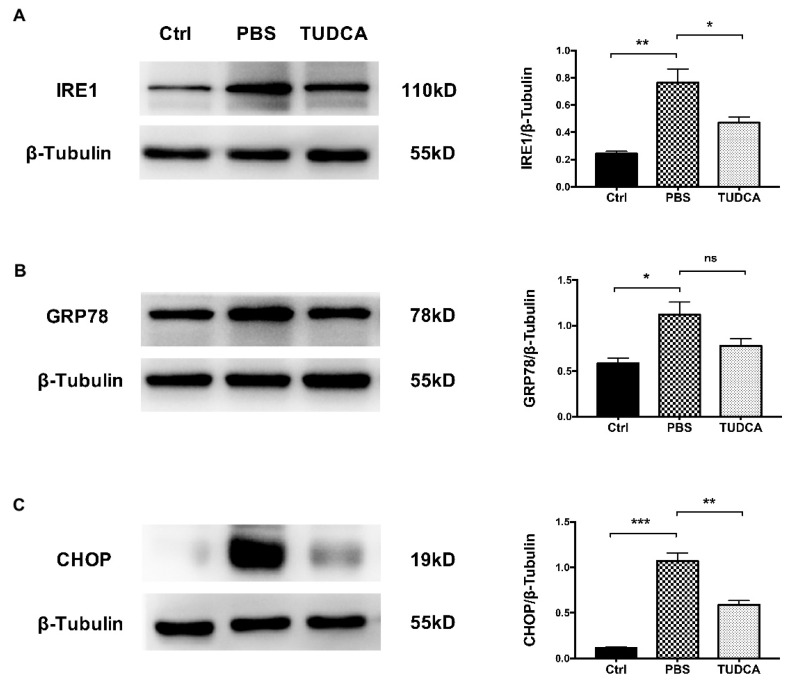
TUDCA inhibits ER stress in OAB retinas. Western blotting analysis of ER stress-related molecules IRE1, GRP78, and CHOP in the retinas of control, PBS-treated, and TUDCA-treated groups on day 1 post-injury. (**A**) Representative gel images and quantified data of IRE1. (**B**) Representative gel images and quantified data of GRP78. (**C**) Representative gel images and quantified data of CHOP. These data are mean ± SEM (*n* = 3, * *p* < 0.05, ** *p* < 0.01, *** *p* < 0.001; ns, no significance). Ctrl, control; OAB, ocular alkali burn; TUDCA, tauroursodeoxycholic acid; GRP78, glucose-regulated protein 78; IRE1, inositol-requiring enzyme-1; CHOP, CCAAT/enhancer-binding protein homologous protein.

We investigated whether the TUDCA treatment could inhibit ER stress in the OAB model. Similar to our previous study, the protein levels of the inositol-requiring enzyme-1 (IRE1), glucose-regulated protein 78 (GRP78), and CCAAT/enhancer-binding protein homologous protein (CHOP) in the retinal tissue were significantly upregulated after the OAB compared to the control group (Figure 7A). However, the TUDCA treatment significantly suppressed the increase in IRE1 and CHOP (Figure 7B–D).

## 3. Discussion

The present study demonstrated that the TUDCA treatment inhibited CNV and promoted corneal re-epithelization. The TUDCA treatment significantly attenuated the expression of proinflammatory factors and the infiltration of CD45-positive cells in OAB corneas. Meanwhile, pretreatment with TUDCA alleviated the apoptosis of RGCs and prevented the structural damage of the retina in OAB eyes. The expression of inflammatory cytokines, the infiltration of CD45-positive cells, and the activation of glial cells in OAB retinas were all reduced after the TUDCA treatment. Our findings suggest that TUDCA inhibits corneal and retinal inflammation and has a protective effect on OAB eyes.

TUDCA is the major bile acid of bear bile and has been recommended by traditional Asian medicine for patients with visual disorders [16]. Previous studies have indicated that TUDCA has anti-apoptotic and anti-inflammatory effects [17,21]. Recently, TUDCA has been shown to provide neuroprotection in several RGC damage models [22,23,24,25]. The research on NMDA-induced RGC degeneration reported that the TUDCA could promote RGC survival against NMDA-induced damage [24]. In a study of the optic nerve crush (ONC) model in rats, TUDCA significantly reduced the apoptosis of RGCs and increased the density of RGCs after ONC [23]. Importantly, Elia et al. evaluated the efficacy and tolerability of TUDCA in patients with amyotrophic lateral sclerosis (ALS) and found that TUDCA was safe and effective in ALS [19]. Moreover, several studies have found that TUDCA has anti-inflammatory effects in neurodegenerative diseases [18,21,26]. In models of retinitis pigmentosa, TUDCA was found to inhibit inflammation and prevent photoreceptor degeneration [21,27]. To our knowledge, in clinical practice, there are few options that can reduce corneal and retinal damage simultaneously. This is the first study to explore the protective effects of TUDCA from both anterior and posterior perspectives. This is the first study to explore the protective effects of TUDCA for both anterior and posterior segments simultaneously.

Damage to the cornea by alkali can lead to an overwhelming inflammatory response and corneal epithelial defects in the acute phase. The rapid re-epithelialization of the defected area is important to reduce the risk of corneal keratolysis and CNV. Successful epithelialization requires an effective resolution to suppress exacerbated inflammation after an OAB [28,29]. After alkali burns, the inflammatory cells are recruited into the burnt cornea and release proinflammatory cytokines, such as IL-1β, IL-6 and TNF-α, which in turn induce the formation of CNV and delay the recovery of corneal epithelium [30,31]. Similar findings were also shown in the present study. The expression of TNF-α and IL-1β in the cornea was significantly increased after the OAB, accompanied by the infiltration of a large number of CD45-positive cells. Previous studies have showed that the inhibition of inflammation can promote wound healing and reduce CNV in OAB corneas [8,28]. In our study, the TUDCA treatment significantly reduced the expression of proinflammatory factors and the infiltration of CD45-positive cells in OAB corneas. We also found that the TUDCA treatment ameliorated CNV and promoted corneal re-epithelization. Our results are in accordance with a previous study showing that TUDCA suppressed laser-induced choroidal neovascularization formation in rats, which might be associated with anti-inflammatory action [32].

In addition to the cornea damage, recent publications reported that OAB could also irreversibly affect the retina and induce RGC loss, which is associated with the upregulation of inflammatory cytokines and activated immune cells in the retina, rather than the pH level or intraocular pressure (IOP) [6,10,33]. Treatment with infliximab (TNF-α inhibitor) could reduce the apoptosis of RGCs after OAB [33]. Our study found that treatment with TUDCA significantly reduced the expression of TNF-α and IL-1β and the infiltration of CD45-positive cells in OAB retinas. We also found that systemic TUDCA treatment attenuated RGC apoptosis and prevented a retinal structure in OAB eyes. The neuroprotective effect of TUDCA was comparable to that of infliximab in the OAB mouse model. These results agree with those of recent studies that reported that TUDCA decreased retinal inflammation by reducing the retinal expression of TNF-α and IL-1β and protected RGCs from cell death in streptozotocin (STZ)-induced diabetic mice [34,35]. These results indicated that TUDCA had anti-inflammation and neuroprotective effects on an OAB retina. Because TUDCA is well-tolerated and has no adverse events in human trials, it can be considered for the clinical treatment of an OAB.

Neuroinflammation, which is characterized by retinal glial activation, has also been considered critical for the pathogenesis of RGC injury [36,37,38,39]. In a previous study, Paschalis et al. found that a peripheral monocyte infiltrated into the retina from the optic nerve at 24 h, and microglia became active at 7 days after an OAB. The prompt administration of anti-TNF-α inhibited the monocyte infiltration and microglia activation, thereby protecting the OAB retina [10]. Our study showed a similar finding that microglia were activated after the OAB, and microglia activation was partly attenuated by the TUDCA treatment. Our findings were also similar to those of recent studies that reported that systemic treatment with TUDCA attenuated changes in the number, distribution, and morphology of microglial cells in a model of retinal degeneration [21]. Furthermore, we observed that GFAP^+^ glial cells were activated after an OAB. The number of GFAP^+^ glial cells peaked on day 3 after an OAB and decreased after TUDCA treatment. These results are consistent with those reported by Bhargava et al. They detected reductions in activated microglia and astrocytes following TUDCA treatment, which ameliorated the neuropathology in a mouse model of multiple sclerosis [40]. These results suggested that the neuroprotective effect of TUDCA might partly be related to the inhibition of neuroinflammation in OAB eyes.

Our previous study found that ER stress is involved in the retinal damage after an OAB. In addition, treatment with 4-PBA (a common ER stress inhibitor) inhibited the expression of ER stress-related molecules and inflammatory factors in the retinal tissue and reduced RGC apoptosis after an OAB [15]. The present study showed that TUDCA, another classical ER stress inhibitor, also decreased the protein levels of IRE1 (the mediator of ER stress-related inflammation) and CHOP (the mediator of ER stress-induced apoptosis) in OAB retinas. Other researchers have also reported that TUDCA inhibits ER stress and has a protective effect in degenerative retinal diseases. In some models of photoreceptor degeneration, TUDCA treatment had a direct effect on photoreceptor survival by reducing ER stress and led to the significant preservation of retinal function [41,42,43]. We speculate that the anti-inflammatory and protective effects of TUDCA may be related to the inhibition of ER stress, but further experiments are needed to confirm this hypothesis.

## 4. Materials and Methods

### 4.1. Animals and OAB Model

Adult male C57BL/6j mice (age: 6–8 weeks, weight: 17–20 g; Laboratory of Zhongshan Ophthalmic Center, Guangzhou, China) were used in this study. All animal procedures were performed in accordance with the Association for Research in Vision and Ophthalmology (ARVO) Statement for the use of animals in research and approved by the Zhongshan Ophthalmic Center Animal Care and Use Committee, Sun Yat-sen University, Guangzhou, China. For OAB modeling, mice were anesthetized with an injection of 1.5% pentobarbital sodium (Sigma-Aldrich, St. Louis, MO, USA). A drop of topical proparacaine was applied to the cornea, followed by a 2.0 mm diameter filter paper that was submerged in 1 M NaOH for 10 s before it was applied to the surface of the right central cornea for 40 s. After alkali exposure, the eye was promptly irrigated with sterile saline solution for 60 s.

### 4.2. Pharmaceutical Intervention

TUDCA (Calbiochem, Merck Millipore, Darmstadt, Germany) was dissolved in sterile phosphate-buffered saline (PBS) solution, followed by adjustment of pH to 7.4 with NaOH. The concentration of TUDCA used in the present study was selected on the basis of previous reports [27,44]. Mice in the TUDCA-treated (400 mg/kg; 250 μL per injection) and PBS-treated (250 μL per injection) groups were injected intraperitoneally (IP) once a day for 3 days prior to establishment of the OAB model. TUDCA was not administrated after OAB. A single injection of Infliximab (Janssen Biotech, Horsham, PA) (6.25 mg/kg) was administered IP immediately after OAB.

### 4.3. Experimental Design

The whole experiment involved 174 C57BL/6j mice. The eyes, corneas, and retinas of all mice were collected at 1 day, 3 days, or 7 days after OAB. The samples were divided into three groups: control group (no OAB and treated with PBS), PBS-treated group (OAB and treated with PBS), and TUDCA-treated group (OAB and treated with TUDCA). The number of mice in the PBS-treated group and the TUDCA-treated group was 78, respectively. Moreover, 18 mice were treated with Infliximab.

### 4.4. Mouse Slit Lamp Examination

Anterior chamber phenotypes were assays and images collected with a slit-lamp digital image processing system (SL-D7/DC-3; Topcon, Tokyo, Japan). CNV was scored as follows: 0 (no neovascularization), 1 (neovascularization in the peripheral cornea, <1/3 area of the corneal), 2 (neovascularization, <2/3 area of the cornea), and 3 (neovascularization, the entire cornea) [45]. Scoring was performed by two independent ophthalmologists who were blinded to the experiment. Corneal epithelial defect areas were detected by fluorescein staining (one drop of 1% fluorescein solution) and calculated using ImageJ software (National Institutes of Health, Bethesda, MD, USA).

### 4.5. Terminal Deoxynucleotidyl Transferase-Mediated dUTP Nick-End Labeling (TUNEL) Assay

Eyes (*n* = 6/group) were collected on day 1 after OAB and fixed in 4% paraformaldehyde at 4 °C overnight. Then, 10% sucrose was used to dehydrate for 1 day and replaced with 20% sucrose for 8 h, followed by 30% sucrose overnight. Afterward, the eyeballs were embedded in optimal cutting temperature compound (Sakura Finetechnical Co., Tokyo, Japan) at −80 °C and sectioned into 10 μm slices through the optic disc. TUNEL staining (in situ Cell Death Detection Kit, TMR red, version 12, Roche, Basel, Switzerland) was performed according to the manufacturer’s instructions. For double staining of TUNEL and Brn3a, sections were first stained with goat polyclonal antibodies against Brn3a (1:500; Santa Cruz Biotechnology, Dallas, TX, USA) at 4 °C overnight, and then incubated with donkey anti-goat IgG H&L (Alexa Fluor 488; 1:500; Abcam, Cambridge, UK) secondary antibody together with TUNEL staining. The retinal sections were then stained with 4,6-diamidino-2-phenylindole (DAPI) and photographed using a confocal microscope (Zeiss LSM800 or Zeiss LSM880).

### 4.6. Histological Analysis

Eyes (*n* = 6/group) were fixed in 4% formalin for 24 h, dehydrated with gradient reagent alcohol, and then embedded in paraffin and cut into 4 μm slices through optic disc. For histological detection, sections were deparaffinized and treated with hematoxylin and eosin solution (H&E). Histological analyses of corneal and retinal tissues were observed using a microscope (Axioplan2; Zeiss, Oberkochen, Germany). All images were processed with ImageJ software.

### 4.7. Immunofluorescence

The frozen sections were blocked with 5% BSA/0.3% Triton X-100 at room temperature for 2 h, and then incubated with primary antibodies against CD45 (1:200; BD Pharmingen, CA, USA), IBA1 (1:500; Wako Pure Chemical Industries, Japan) or GFAP Cy3 Conjugate (1:500; Sigma-Aldrich, USA) at 4 °C overnight. After washing three times with PBS, sections were incubated with secondary antibodies conjugated with Alexa Fluor 488 (1:500; Abcam) or Alexa Fluor 647 (1:500; Abcam) for 2 h at room temperature, stained with DAPI, and photographed using a confocal microscope (Zeiss LSM800 or Zeiss LSM880).

For flat-mounted retina, the eyeballs (*n* = 6/group) were enucleated 7 days after OAB and fixed in 4% paraformaldehyde for 50 min. The whole retinas were carefully prepared and permeabilized with 0.3% Triton X-100 and blocked with 5% BSA overnight. Next, they were incubated with primary antibodies against Brn3a (1:500; Santa Cruz Biotechnology) or IBA1(1:500; Wako Pure Chemical Industries) for 24 h and washed in PBS, and then incubated with secondary antibody conjugated with Alexa Fluor 488 (1:500; Abcam). After three more PBS washes, DAPI was used to stain sections for 10 min. Finally, retinas were mounted with anti-fade mounting medium. All images were collected by a confocal microscope (Zeiss LSM800 or Zeiss LSM880).

### 4.8. Reverse-Transcriptase Polymerase Chain Reaction (RT-PCR)

Retinas (*n* = 6/group) were harvested 1 day after OAB, and corneas (*n* = 6/group) were collected 7 days after OAB. Total RNA was extracted from mice retinas or corneas with TRIzol (Takara, Kustatsu, Japan) and then reversely transcribed to cDNA using PrimeScript^TM^RT reagent kit (Takara). The SYBR Green I RT-PCR Master mix was used according to the manufacturer’s protocol. The PCR primers (Table 1) were designed based on the NCBI GenBank database.

Quantitative PCR was measured by the LightCycler 480 II system (Roche). The PCR amplification protocols consisted of 95 °C for 5 min, followed by 40 cycles of 95 °C for 5 s and 60 °C for 15 s. GAPDH was used as a reference gene and subjected to calculate target gene expression by the 2-ΔΔCq method.

### 4.9. Enzyme-Linked Immunosorbent Assay (ELISA)

Retinas (*n* = 6/group) were dissected 1 day after OAB, and corneas (*n* = 6/group) were collected 7 days after OAB. The corneas or retinas of two mice per group were obtained and homogenized in lysis buffer (KeyGen Biotech, Nanjing, China) including protease and phosphatase inhibitors. Total protein levels of TNF-α and IL-1β were measured by ELISA kits (Cat# TNF-α: 88-7324-88; IL-1β: 88-7013-88; Invitrogen, Thermo Fisher Scientific, USA), according to the manufacturer ’s instructions.

### 4.10. Western Blotting

Retinas were dissected 1 day after OAB. Whole protein was extracted from mice retinas with lysis buffer (KeyGen Biotech, Nanjing, China) including protease and phosphatase inhibitors. The protein concentrations were quantified with a Pierce^TM^ BCA Protein Assay Kit (Thermo Fisher Scientific, Waltham, MA, USA). Equal amounts of protein from each sample were loaded into lanes on a 10% sodium dodecyl gel for polyacrylamide gel electrophoresis and then transferred to polyvinylidene difluoride membranes (Bio-Rad, Hercules, CA, USA). The membranes were blocked in 5% skim milk in Tris-buffered saline containing Tween (TBST) for 2 h and then incubated with primary antibodies against IRE1 (1:1000, Abcam), GRP78 (1:1000, BD Biosciences, Franklin Lakes, NJ, USA), CHOP (1:1000, Abcam), or β-tubulin (1:1000, Cell Signaling, Danvers, MA, USA) at 4 °C overnight. Then, the membranes were washed three times with TBST and incubated with secondary antibodies (1:5000, Abcam) for 1 h at room temperature. The protein bands were visualized on a ChemiDoc Touch Imaging System (Bio-Rad), and band intensity was analyzed using ImageJ software.

### 4.11. Quantitative and Statistical Analyses

Cell numbers in the corneal or retinal tissue were counted by two members of our team who were blinded to the group using ImageJ software. Three images were taken from each mouse, with 6 mice in each group, and a total of 18 images were used to count the number of cells in the corneal or retinal tissue. Average cell counts from three images per mouse were used for statistical analysis. For the analysis of macroglia activation, GFAP^+^ fibers crossing the inner plexiform layer (IPL) and the inner nuclear layer (INL) were quantified. All data are presented as mean ± SEM. Comparisons of two or three groups at different time points were analyzed using two-way analysis of variance followed by Bonferroni’s multiple comparison test. Comparisons of three or four groups were analyzed using one-way analysis of variance followed by Bonferroni’s multiple comparison test. Statistical analysis was performed using commercial statistical software (Prism; GraphPad, Inc., La Jolla, CA, USA). *p* < 0.05 was considered significant.

## 5. Conclusions

In conclusion, we demonstrated that TUDCA inhibited the corneal and retinal inflammation induced by an OAB. Systemic TUDCA treatment not only ameliorated CNV and promoted corneal re-epithelization but also attenuated RGC apoptosis and preserved the retinal structure after an OAB.

## Figures and Tables

**Table 1:** Primer sequences of mice TNF-α, IL-1β, and GAPDH for RT-PCR.

Gene	Forward	Reverse
*TNF-α*	TGCCTATGTCTCAGCCTCTT	GAGGCCATTTGGGAACTTCT
*IL-1β*	TAGAGCTGCTGGCCTTGTTA	ACCTGTAAAGGCTTCTCGGA
*GAPDH*	TGCACCACCAACTGCTTAG	GGATGCAGGGATGATGTTC

*TNF-α*, tumor necrosis factor alpha; *IL-1β*, interleukin-1 beta; and *GAPDH*, glyceraldehyde-3-phosphate dehydrogenase.

## Data Availability

All relevant data is contained within the article.
